# Isthmin 2 is decreased in preeclampsia and highly expressed in choriocarcinoma

**DOI:** 10.1016/j.heliyon.2020.e05096

**Published:** 2020-10-13

**Authors:** Cynthia Martinez, Javier González-Ramírez, María E. Marín, Gustavo Martínez-Coronilla, Vanessa I. Meza-Reyna, Rafael Mora, Raul Díaz-Molina

**Affiliations:** a Department of Obstetrics and Gynecology, Maternity and Children Hospital, Mexicali, B.C., 21376, Mexico; b Faculty of Nursing, University Autonomous of Baja California, Mexicali, B.C., 21100, Mexico; c School of Medicine, University Autonomous of Baja California, Mexicali, B.C., 21000, Mexico; d Laboratory of Surgical Pathology and Cytodiagnosis, Mexicali, B.C., 21389, Mexico

**Keywords:** Pregnancy, Obstetrics and gynecology, Reproductive system, Evidence-based medicine, Laboratory medicine, Biomarkers, ISM2, Preeclampsia, Choriocarcinoma, Angiogenic protein

## Abstract

**Introduction:**

Isthmin 2 (ISM2) is a protein which expression in humans is almost specific to the placenta. There is no previous report in the literature that investigated this protein in preeclampsia or choriocarcinoma.

**Methods:**

We conducted a prospective, cross-sectional study that included women with preeclampsia, gestational hypertension and normotensive pregnancy. We measured serum concentrations of ISM2 protein and performed immunohistochemistry in placenta tissues. We also performed immunohistochemistry of ISM2 in samples from choriocarcinoma and compare with lung, prostate, colon, gastric and breast cancers.

**Results:**

A total of 81 patients were included, 30 with preeclampsia, 21 with gestational hypertension and 30 controls. The ISM2 protein was found to be decreased in patients with preeclampsia compared to the control group (P = 0.036). These results were confirmed by immunohistochemistry. We also found that ISM2 protein was overexpressed in choriocarcinoma.

**Discussion:**

Taken together, our results suggest an angiogenic function for ISM2. Its serum level decreased in our patients with preeclampsia could be reflecting that it is involved in the pathogenesis of the disease; on the other hand its high expression in choriocarcinoma, indicates that ISM2 may play an active role in the angiogenesis of this and other cancers.

## 1 Introduction

Preeclampsia is a major disease in the field of obstetrics. It has remained as a potentially life-threatening condition for pregnant women in both developed and developing countries [1]. Worldwide its incidence is 5–18% [2, 3, 4, 5, 6], contributing to an estimate of 70,000 maternal deaths and 500,000 perinatal deaths annually on the planet [7]. The etiology of preeclampsia is unknown, however, the most accepted theory is that the placenta is the primary organ in its pathogenesis since only removal of the placenta suppresses the disease [6]. In fact, only the placenta and not the fetus is required for its development [8, 9, 10]. Many lines of research continue to support the hypothesis that preeclampsia results from liberation of placental factors to the circulation, which in turn leads to maternal endothelial dysfunction [11]. From this perspective, the disease reflects widespread endothelial dysfunction, due to an imbalance of angiogenic and antiangiogenic factors [12, 13]. Dozens of studies have shown that women with preeclampsia have high levels of circulating antiangiogenic proteins such as the soluble fms-like tyrosine kinase-1 (sFlt1), soluble endoglin (sEnd) and low levels of angiogenic proteins as the vascular endothelial growth factor (VEGF) and placental growth factor (PlGF) [12, 13, 14, 15, 16, 17, 18].

In contrast, choriocarcinoma a very aggressive neoplasm originated from trophoblastic cells of the placenta is characterized by highly pro-angiogenic with rapidly growing rate and metastatic malignant epithelial tumors [19]. The angiogenic proteins, as VEGF and PlGF are overexpressed in these tumors [20, 21], whereas sFlt1 expression is strongly suppressed [22]. Incidence of choriocarcinoma has been reported 1 in 24,096 pregnant patients [23]. Most cases arise following gestational events but also exist the non-gestational types in males or females as lung, gastric, colon, ovarian and testis cancer [24, 25, 26].

Isthmin proteins represent a new family of secreted proteins [27, 28]. The name isthmin makes reference that the first member of this family was described by its high expression in the brain of Xenopus, in a region called isthmus [27]. It was therefore named isthmin. In the human genome, there are two isthmin genes [isthmin 1 (ISM1) and isthmin 2 (ISM2)], both of which encode secreted proteins that exhibit signal peptides, as well as thrombospondin-1 (TSR1) and Adhesion-associated domain in MUC4 and Other Proteins (AMOP) domains. While the ISM1 gene encodes for a protein of ~50 kDa, the ISM2 gene encodes for a protein of ~63.9 kDa [29]. Because of their structure, previous studies focused on the potential role of ISM1 in angiogenesis, finding that it is a protein with both angiogenic and anti-angiogenic activity [30, 31, 32]. We recently published the expression of ISM1 in humans and showed that one of the sites of greatest expression is the placenta [29]. Expression data indicated that ISM2 expression in humans is almost specific to the placenta [28, 29], however there is no previous work conducted to elucidate the potential function of ISM2. In this study we were interested in assessing whether ISM2 protein is dysregulated in preeclampsia and choriocarcinoma, considering that both diseases involved placenta dysfunction.

## 2 Materials and methods

### 2.1 Study population

We conducted this prospective cross-sectional study at the Maternity and Children Hospital of Mexicali, Baja California, Mexico, from August 2016 to March 2017. The study was approved by the Local Ethics Committee (Register number 02-01-HGMXL/GO-2015-11-23/124). A written informed consent to participate in the study was obtained from all of the participants.

Inclusion criteria were women with singleton pregnancies, no history of any disease and diagnosis of preeclampsia or gestational hypertension. At first, the patients were diagnosed as hypertensive disorder in pregnancy as long as laboratory or clinical evolution was needy to classify them into one of the category group: preeclampsia or gestational hypertension. The criteria diagnosis were based on the “ACOG, Task Force on Hypertension in Pregnancy 2013” criteria [33]: women known to be normotensive who developed a systolic blood pressure (SBP) ≥140 mmHg or diastolic blood pressure (DBP) ≥ 90 mmHg on 2 occasions at least 4 h apart after the 20th week gestational age and proteinuria ≥300 mg/24 h urine collection. In the absence of proteinuria, preeclampsia was diagnosed as hypertension with new onset of thrombocytopenia (platelet count <100 × 10^3^/μL), elevated liver transaminases (twice the normal range), renal insufficiency (creatinine level >1.1 mg/dL), pulmonary edema and/or new onset of cerebral or visual disturbances. Any patient with blood pressure ≥160/110 mmHg on 2 occasions persistently or 4 h apart was classified as preeclampsia. Exclusion criteria were fetuses with chromosomal or congenital anomalies and stillborn. Clinical data were ascertained prospectively including laboratory values. All pregnancy outcomes were recorded.

### 2.2 Measurement of circulating ISM1 and ISM2 concentration

Given that ISM2 belongs to the Isthmin Family of proteins, we measured ISM1 in order to know its pattern of expression as internal control. Serum samples from patients and controls were used for the quantification of the proteins ISM1 and ISM2 using enzyme-linked immunosorbent assays (ELISA). For the determination of ISM1 in serum, the procedure was performed with the use of a commercial kit (Cloud-Clone Corp., Katy, TX, USA). The kit is a sandwich enzyme immunoassay for in vitro quantitative of ISM1. According to manufacturer, intra-assay coefficient of variation (CV) is less than 10%, inter-assay CV is less than 12% and the minimum detectable dose of ISM1 is less than 0.066 ng/mL. For the determination of ISM2 in serum, the procedure was performed using a commercial kit (MyBioSource Inc., San Diego, CA, USA). It is a quantitative sandwich ELISA kit using purified human ISM2 antibody to coat strip plate wells. According to manufacturer, intra and inter-assay CV is less than 15% and sensitivity is 5.0 pg/mL.

### 2.3 Immunohistochemistry of ISM1 and ISM2

Placenta tissue was obtained after the delivery from controls and patients. Choriocarcinoma and cancer samples were obtained from our pathology archives of paraffin-embedded blocks. Immunohistochemistry was performed with primary anti-ISM1 and anti-ISM2 antibodies (MyBiosource Inc., San Diego, CA, USA), using the Mouse/Rabbit PolyDetector Plus HRP/DAB Polymeric Detection System with microarray controls of tissue processed in the laboratory. To confirm the reaction specificity of the antibody, internal and external, negative and positive controls were used. Briefly, human placental and cancer sections were de-paraffinized in xylene and rehydrated in a series of graded alcohols. After quenching the activity of endogenous peroxidase with 1% H_2_O_2_ in PBS for 10 min, the sections were rinsed three times with PBS and then incubated with 5% non-fat milk/PBS for 30 min to reduce non-specific bindings. Sections were incubated with primary antibodies, an anti-ISM1 polyclonal antibody or anti-ISM2 polyclonal antibody (MyBiosource Inc., San Diego, CA, USA). The primary antibody was incubated for 10 min using a polymer based visualization kit according to the manufacturer's instructions; once the reaction was complete, it was revealed with diaminobenzidine and counterstained with hematoxylin and examined under a light microscope. Negative controls were performed by replacing primary antibodies with an isotype control IgG. A pathologist evaluated any positivity in the tissue sections and sites and cells stained positive were recorded. The staining intensity of ISM2 marker in cancer samples was scored as 0, 1+, 2 + or 3+, and the percentage of immunoreactive cells were documented. All of the placenta samples were match with the serum samples.

### 2.4 Statistical analysis

Since this was an exploratory study, no formal power analysis to determine sample size was performed. Characteristics of patients were summarized as medians (quartile 1, quartile 3) and compared by using of Kruskal-Wallis test. Serum levels of ISM1 and ISM2 were presented as medians (quartile 1, quartile 3) and compared by using of Mann-Whitney U test. Values of P < 0.05 were considered to indicate statistical significance.

## 3 Results

### 3.1 Characteristics of the participants

A total of 81 patients were analyzed. Among them 30 had diagnosis of preeclampsia, 21 were diagnosed with gestational hypertension and 30 women served as healthy pregnant controls. The clinical and laboratory characteristics of all of the enrolled patients are presented in Table 1. Although there was no significant difference in age, body mass index (BMI), gravida or parity, we found five patients with preeclampsia weighted 110–117 kg, four patients with gestational hypertension weighted 100–108 kg, and only one control was 104 kg weight.

**Table 1:** Maternal, perinatal and laboratory characteristics in all of the groups.

Variable	Preeclampsia∗ N = 30	Gestational Hypertension∗ N = 21	Normotensive Pregnancy∗ N = 30	P
Age [years]	20.5 (19–27)	19 (18–22)	21.5 (20–25)	0.17
Weight [kg]	86.9 (72–98)	87.7 (68.7–101.8)	78.2 (69–85.4)	0.07
BMI [kg/m^2^]	35.4 (29.6–37.5)	35.5 (28–39.1)	30.5 (28.7–33.3)	0.05
Gravida	1 (1–3)	1 (1–2)	2 (1–2)	0.43
Gestational age [WG]∗∗	38.2 (37.1–39.5)	39.2 (38.7–40.3)	39.3 (38.4–40.1)	0.70
Receipt of any antihypertensive	18 [60%]	7 [33%]	0	-
Receipt of MgSO_4_	3 [10%]	0	0	-
Eclampsia	1 [3.3%]	0	0	-
Parity	0 (0–1)	1 (0–1)	1 (0–2)	0.53
SBP [mmHg]	150 (140–160)	140 (130–147)	110 (102–116)	0.00
DBP [mmHg]	100 (100–110)	90 (90–100)	70 (66–76)	0.00
Neurologic symptoms	6 [20%]	0	0	-
CS delivery	18 [60%]	6 [28%]	10 [33.3%]	-
Birth weight [g]	2810 (2470–3340)	3220 (2880–3520)	3315 (2950–3590)	0.01
Capurro score	38.2 (37.2–39.5)	39.1 (39–40.3)	39.6 (38.4–40.1)	0.70
Preterm birth	3 [10%]	0	0	-
Male sex	18 [60%]	12 [57.1%]	9 [30%]	-
Hemoglobin [g/dL]	12.2 (11.6–13.5)	12.3 (11.9–12.8)	12.6 (11.7–13.6)	0.74
Hematocrit [%]	37.5 (34.6–40.3)	38 (36.4–39.8)	39.1 (35.5–40)	0.83
Platelets [10^3^/μL]	200 (151–235)	199 (162–223)	228 (201–260)	0.83
AST [U/L]	17 (14–26)	16 (12–18)	16 (14–21)	0.50
ALT [U/L]	12 (8–18)	10 (14)	10 (8–16)	0.72
Total bilirubin [mg/dL]	0.3 (0.3–0.4)	0.4 (0.3–0.4)	0.5 (0.3–0.6)	0.02
LDH [U/L]	327 (282–409)	299 (272–356)	322 (261–355)	0.65
Creatinine [mg/dL]	0.6 (0.5–0.7)	0.6 (0.5–0.6)	0.5 (0.5–0.6)	0.00
Urea [mg/dL]	17.1 (12.8–21.4)	17.1 (6.4)	12.8 (4.2)	0.01
BUN [mg/dL]	8 (6–10)	8 (6–9)	6 (10–15)	0.02
Uric acid [mg/dL]	4.9 (4.3–6.3)	4.6 (3.9–5.5)	3.8 (3.1–4.5)	0.00
Glucose [mg/dL]	87 (75–97)	84 (77–99)	89 (82.5–96.5)	0.78
Urine protein [mg/24 H]	972 (425–1652)	152 (97–190)	-	0.00
WBC [10^3^/μL]	10.4 (3.51)	9.0 (7.7–10.5)	9.4 (8.5–11.7)	0.33
PT [s]	10.4 (0.9)	10.9 (10.5–11.3)	11.1 (10.6–11.4)	0.00
PTT [s]	25.7 (6)	27.4 (23.2–28.1)	26.7 (25.6–29.7)	0.30
ISM1 [ng/mL]	2.5 (2–3.2)	2.8 (2.4–3)	2.6 (2–3)	0.68
ISM2 [pg/mL]	51.7 (27.9–86.2)	82.6 (26.9–125.3)	110 (32.6–209.4)	0.03

Abbreviations: N, number. BMI, body mass index. SBP, systolic blood pressure. DBP, diastolic blood pressure. CS, cesarean section. AST, aspartate aminotransferase. ALT, alanine aminotransferase. LDH, Lactate dehydrogenase. BUN, blood urea nitrogen. WBC, white blood cells. PT, Prothrombin Time. PTT, partial thromboplastin time. H, hours. s, seconds. ISM1, isthmin 1. ISM2, isthmin 2. Q1, quartile 1. Q3, quartile 3. WG, weeks of gestation.

Kruskal-Wallis test and Mann Whitney U test were used.

Statistical significance: P < 0.05.

∗ Medians are presented and Q1-Q3 are shown in parentheses or N [%].

∗∗ Gestational age at sample collection. The difference between the sample collection and delivery was 2 h–7 days.

As expected, there was significant difference in systolic or diastolic blood pressures among the three groups. The gestational age at the sample collection was a median of 37 weeks of gestation (WG) in preeclampsia group, 39.1 WG in gestational hypertension patients and 39.3 WG in controls. However, three samples of preeclampsia patients correspond to preterm pregnancy: 33.3, 33.1 and 29.6 WG (not reflected in Table 1, because it shows the median and interquartile range).

Six (20%) patients of the preeclampsia group referred neurological manifestations (e.g. headache), and one patient presented eclampsia immediately after she arrived to emergency service. In regard to treatment, 60% of patients with preeclampsia received some antihypertensive before delivery (e.g. nifedipine, methyldopa, hydralazine) compared with 33% of gestational hypertension patients. Additionally, 10% of patients in preeclampsia group were giving an infusion of magnesium sulfate (MgSO_4_).

Frequency of delivered by cesarean section was higher in preeclampsia group (60%) compared with the other groups (P = 0.03). Women with preeclampsia had lower birth weight babies; the lowest were 1270, 1940 and 2030 g. On the contrary, the lowest birth weight in gestational hypertension group was 2740 g and in control group was 2870 g. Male sex newborn was the predominant in preeclampsia and gestational hypertension (60% and 57.1% respectively), while controls had majority female babies (70%).

The difference between the sample collection and delivery was about 2 h–7 days, which relied on gestational age, pulmonary maturation needy, labor induction and evolution of patients (deterioration or steady clinical condition).

Patients with preeclampsia had higher levels of creatinine, uric acid, blood urea nitrogen (BUN) and proteinuria. The higher proteinuria values were 4817, 3049, 2986 and 2042 mg/24 h urine collection in our patients with preeclampsia, and for definition all our patients with gestational hypertension presented proteinuria less than 300 mg/24 h urine collection.

Three of our preeclampsia patients exhibited platelet count <100 × 10^3^/μL but this did not represent significant difference between the groups. Similarly, lactate dehydrogenase (LDH), bilirubin and aminotransferases levels, showed no significant difference between the groups, although some of our preeclampsia patients had elevated aminotransferases.

### 3.2 ISM1 is not dysregulated in preeclampsia

**Figure 1:**
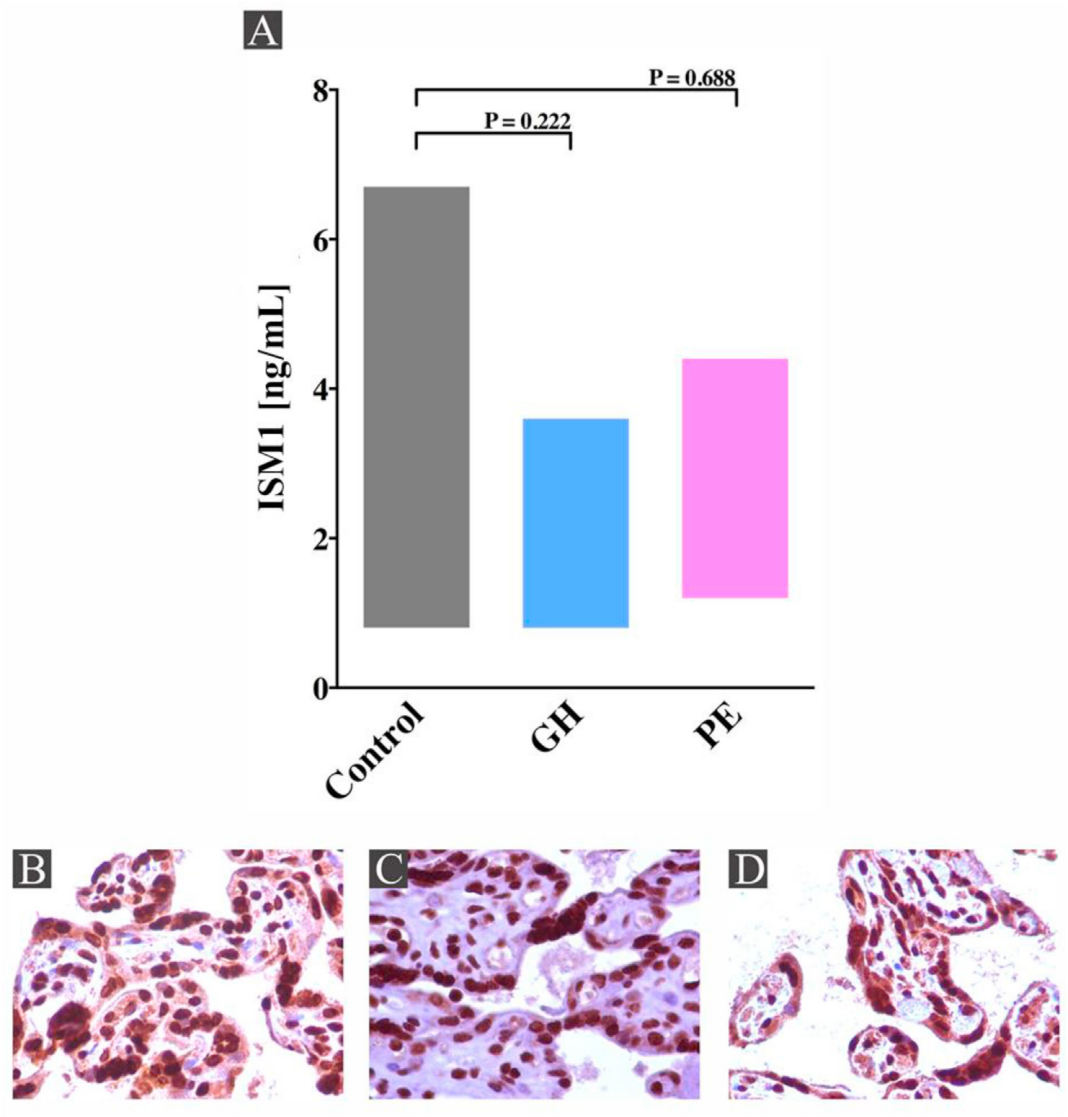
**ISM1 protein shows no difference in the circulation and placenta in patients with preeclampsia.** Circulating ISM1 protein was measured in 30 women with normotensive pregnancy (Control), 21 patients with gestational hypertension (GH) and 30 patients with preeclampsia (PE). In Panel A, ISM1 protein showed no statistically difference in preeclampsia compared with control group and gestational hypertension. We performed immunohistochemistry of ISM1 in placenta tissues from normotensive, gestational hypertension and preeclampsia pregnancies. In Panel B, we observed that 100% of the trophoblastic cells in the normal placentas show diffuse nuclear positivity for ISM1 antibody with accentuated intensity. In Panel C, placentas from gestational hypertension displayed high intensity for ISM1 antibody in about 97% of the trophoblastic cells and finally in preeclamptic placentas ISM1 antibody exhibited strong intensity in 95% of the trophoblastic cells (Panel D). Regarding immunohistochemistry of ISM1, nonrelevant difference between the staining of the ISM1 protein in normal, gestational hypertension and preeclamptic placentas was demonstrated.

The serum levels of ISM1 protein were measured in 30 patients with normotensive pregnancy, 21 patients with gestational hypertension and 30 patients with preeclampsia. The level of ISM1 in the control group was 0.8–6.7 ng/mL (median 2.6 ng/mL), in the gestational hypertension group was 0.8–3.6 ng/mL (median 2.68 ng/mL) and in the preeclampsia group was 1.2–4.4 ng/mL (median 2.5 ng/mL). We compared the medians of ISM1 levels among the three groups and found no statistically significant difference (P > 0.05) (Figure 1A, Table 1). We performed immunohistochemistry of ISM1 in placenta tissues from normotensive, gestational hypertension and preeclampsia pregnancies and confirmed the serum results. We observed that 100% of the trophoblastic cells in the normal placentas show diffuse nuclear positivity for ISM1 antibody with accentuated intensity (Figure 1-B). Placentas from gestational hypertension displayed high intensity for ISM1 antibody in about 97% of the trophoblastic cells (Figure 1-C), and in preeclamptic placentas ISM1 antibody exhibited expression in 95% of the cells with strong intensity (Figure 1-D). We compared the immunohistochemistry of ISM1 and found that nonrelevant difference was exhibited among the staining in normal, gestational hypertension and preeclamptic placentas.

### 3.3 ISM2 is decreased in patients with preeclampsia

**Figure 2:**
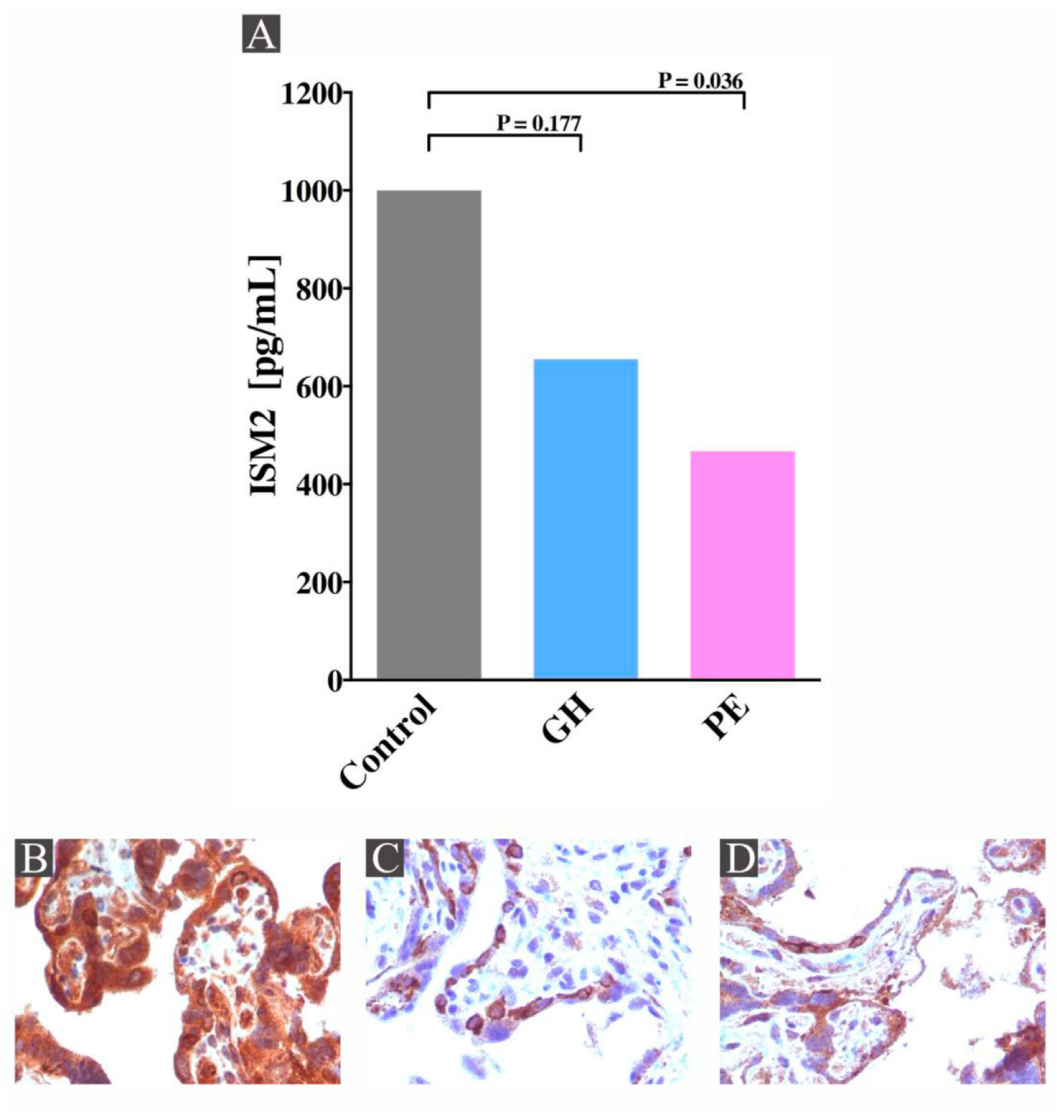
**ISM2 protein is decreased in the circulation and placenta in patients with preeclampsia.** Circulating ISM2 protein was measured in 30 women with normotensive pregnancy (Control), 21 patients with gestational hypertension (GH) and 30 patients with preeclampsia (PE). In Panel A, circulating ISM2 was significantly decreased in women with preeclampsia relative to healthy controls (P = 0.036). We performed immunohistochemistry of ISM2 in placenta tissues from normotensive, gestational hypertension and preeclampsia pregnancies. ISM2 protein expression through immunohistochemistry was also decreased in the preeclampsia group relative to controls. ISM2 showed focal positivity in the cytoplasmic edge in a median of 173 trophoblastic cells per 10 high power fields (HPF) in normal placentas (Panel B), focal positivity in the cytoplasmic edge in a median of 145 trophoblastic cells per 10 HPF in placentas from gestational hypertension (Panel C), and focal positivity in the cytoplasmic edge in a median of 52 trophoblastic cells per 10 HPF in preeclamptic placentas (Panel D).

The serum levels of ISM2 protein were measured in 30 patients with normotensive pregnancy, 21 patients with gestational hypertension and 30 patients with preeclampsia. The level of ISM2 in the control group was 3.5–1000 pg/mL with a median of 110 pg/mL and in the preeclampsia group was 3.5–467.2 pg/mL with a median of 51.7 pg/mL (P = 0.036). The level of ISM2 in the gestational hypertension group was 3.5–655.2 pg/mL with a median of 82.6 pg/mL. Circulating ISM2 was only statistically significant decreased in women with preeclampsia compared with the control group (Figure 2A, Table 1). This result was confirmed by immunohistochemistry. We observed a cytoplasmic positivity for ISM2 with difference in the immunohistochemical staining whether the tissue was derived from preeclampsia, gestational hypertension or control group. To determine the difference, we counted the number of positive immunolabeled cells over the total cells in each selected area. In normal placentas, focal positivity in the cytoplasmic edge was observed for ISM2 in a median of 173 trophoblastic cells per 10 high power fields (HPF) (Figure 2B). In placentas from patients with gestational hypertension we observed a focal positivity in the cytoplasmic edge in a median of 145 trophoblastic cells per 10 HPF (Figure 2C) and in preeclamptic placentas the median of trophoblastic cells stained were 52 cells per 10 HPF (Figure 2D). The positive and negative controls for each of the immunoreactions were corroborated.

### 3.4 ISM2 is highly expressed in choriocarcinoma

We selected from our pathology archives of paraffin-embedded blocks sections of choriocarcinoma and compared with lung, breast, prostate, colon and gastric cancers. We stained the sections with anti-ISM2 antibody.

**Figure 3:**
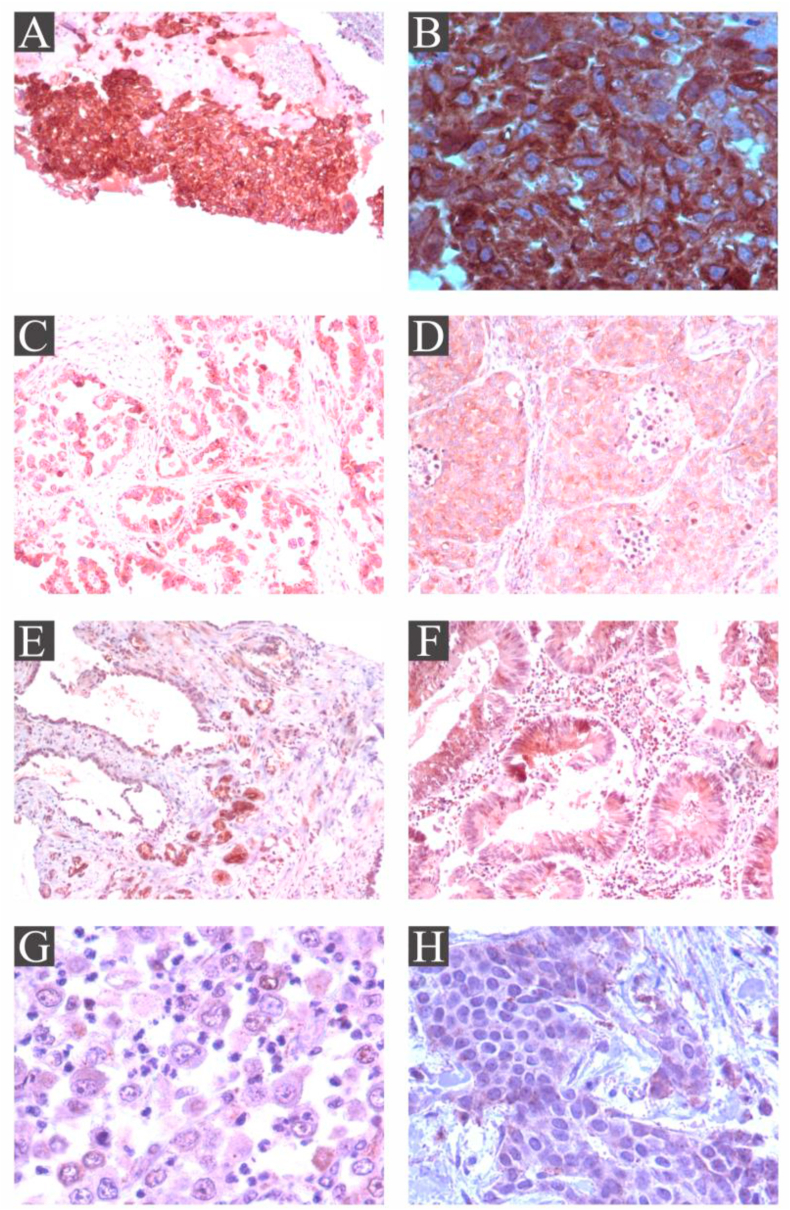
**ISM2 protein is highly expressed in choriocarcinoma compared with lung, prostate, colorectal, gastric and breast cancer tissues.** Tissue sections of lung, prostate, colorectal, gastric and breast cancer tissues were compared with choriocarcinoma. We organized the pictures from the higher expression (scored as 3+, 2+) to lesser expression (1+, 0). In Panels A and B, we displayed two magnification of ISM2 protein staining in choriocarcinoma, Panel A x10, Panel B x40. They show a strong (3+) and diffuse positivity expression of ISM2 protein in all the cancer tissue. In Panels C and D, we can notice that ISM2 protein showed less positivity expression than previous, we observed moderate (2+) and diffuse positivity expression in lung adenocarcinoma (x10). Similarly, adenocarcinoma of prostate showed a moderate (2+) and diffuse positivity expression for ISM2 protein which is depicted in Panel E (x4). Panel F displays a mild (1+) and focal positivity expression for ISM2 protein in colorectal adenocarcinoma (x10). Panel G shows a mild (1+) and focal positivity expression for ISM2 protein in poorly cohesive carcinoma gastric (x40). Panel H displays that the immunoreaction of ISM2 protein was negative in invasive ductal carcinoma of the breast (x10).

We observed strong (3+) and diffuse positivity in choriocarcinoma (Figure 3A,B). On the contrary the expression in all of other cancers was lesser; we found a moderate (2+) and diffuse positivity expression of ISM2 protein in lung adenocarcinoma (Figure 3C,D) and in prostate adenocarcinoma (Figure 3E), mild (1+) focal positivity in colon adenocarcinoma (Figure 3F), mild focal positivity in poorly cohesive carcinoma gastric (Figure 3G), and there was negativity in invasive ductal carcinoma of the breast (Figure 3H). Positive and negative controls were corroborated in each of the cases. A minimum of three experiments per case was made.

## 4 Discussion

This is the first study conducted to evaluate the ISM2 protein in preeclampsia and choriocarcinoma, a protein with almost specific expression in placenta. We showed that patients with preeclampsia have decreased serum levels of ISM2 protein in relation to normal pregnancy and gestational hypertension. This result was statistically significant. On the contrary, we found that ISM2 shows a strong expression in choriocarcinoma. No previously published work of this protein can be found. The two contrasting results of ISM2 protein (decreased in preeclampsia and high expression in choriocarcinoma) could have exciting implications. Preeclampsia is characterized by an imbalance between angiogenic proteins in favor of antiangiogenic proteins [12, 13]. A number of previous studies have shown that antiangiogenic factors such as sFlt-1 and sEng are elevated in preeclampsia, whereas angiogenic factors such as VEGF and PlGF are diminished [12, 13, 14, 15, 16, 17, 18]. They have shown to have potential as biomarkers in this disorder and have suggested angiogenesis deficiency produce the distinctive manifestations of preeclampsia. Contrary to preeclampsia, choriocarcinoma is vastly angiogenic and produces high levels of PlGF to promote the development of blood vessels [20]. Remarkably, although choriocarcinoma is part of a spectrum of conditions known as gestational trophoblastic disease: complete and partial hydatidiform mole, invasive mole, choriocarcinoma and placental-site trophoblastic tumor, they show different pattern of angiogenesis expression, for example, while molar gestations produce more anti-angiogenic proteins similar to preeclampsia [46], expression of sFlt-1 is strongly suppressed in choriocarcinoma cell lines and VEGF is highly expressed in these cells [47]. Indeed, choriocarcinoma can even be developed in males, and therefore not related to the increased in preeclampsia as molar gestations do.

It is widely known angiogenesis is a key factor in the progression of cancer since tumor growth and metastasis depend on it. Upregulation of PlGF expression also occurs during the development of other human malignancies, in fact PlGF has emerged as a valid target for anti-angiogenic therapy [20]. Interestingly, cancer patients receiving anti-angiogenic therapy present hypertension and proteinuria along with glomerular damage as adverse effects that resembling preeclampsia [34, 35, 36].

The structure of ISM2 protein is particularly interesting and its domain combination suggests an angiogenic activity. On one hand, the AMOP domain localized in its extreme C-terminal has been described only in four proteins of the genome: MUC4, SUSD2, ISM1, and ISM2 [28,30,37]. The evidence indicates that the former three are important elements in angiogenesis: MUC4 is a key factor in mediated tumor angiogenesis and metastasis of pancreatic cancer [37, 38, 39], SUSD2 promotes tumor angiogenesis in breast cancer [40], and ISM1 promotes angiogenesis depending on its physical state in vivo [31].

On the other, TSP-1 central domain in ISM2 is part of a vascular regulators family that can be both pro-angiogenic and anti-angiogenic [41]. TSP-1 acts as an angiogenic factor to promote endothelial cell proliferation, migration, and overall vascular growth, as well has a critical role in follicular angiogenesis [41, 42, 43, 44]. Moreover, TSP-1 also works as a cancer promoter in many different and complex pathways [45].

From a clinical perspective, our findings could help to a better diagnosis and prediction of preeclampsia if we are able in next studies to reveal the differences of expression of ISM2 before the onset of preeclampsia and at the disease stage. On the other hand, in upcoming researches to compare the levels of ISM2 with angiogenic factors already described in preeclampsia as PlGF, and VEGF, is a mandatory next stage.

Nothing is known about the three potential isoforms for ISM2 that exists; and although all of them are secreted proteins according to The Human Gene Database [https://www.genecards.org/], further exploration should reveal a better understanding of the functional relationship between these isoforms.

We conclude that ISM2 is a protein with angiogenic function, which shows under normal conditions its highest expression in placenta and decreases in pregnant women with preeclampsia. In order to modulate disease progression, we need to consider another way to treat preeclampsia, a treatment which targets were angiogenic factors (to increase them) or antiangiogenic factors (to blocked them) since the evidence continues showing the relevance of these factors in the pathology of the disease. In addition, ISM2 may also play a role in the angiogenesis of choriocarcinoma. Our findings open to new studies that allow us to elucidate the participation of ISM2 in the development of preeclampsia, and moreover to characterize its potential angiogenic function in choriocarcinoma and other cancers.

## Declarations

### Author contribution statement

C. Martinez and R. Díaz-Molina: Conceived and designed the experiments; Performed the experiments; Analyzed and interpreted the data; Contributed reagents, materials, analysis tools or data; Wrote the paper.

M. Marin: Conceived and designed the experiments; Performed the experiments; Analyzed and interpreted the data.

V. Meza-Reyna: Performed the experiments.

J. González-Ramírez and G. Martínez-Coronilla: Contributed reagents, materials, analysis tools or data.

### Funding statement

This work was supported by University Autonomous of Baja California, México, second special call for support to research projects, grant number 1233.

### Competing interest statement

The authors declare no conflict of interest.

### Additional information

No additional information is available for this paper.
